# Characterization of the Bacterial Community Associated with Larvae and Adults of *Anoplophora chinensis* Collected in Italy by Culture and Culture-Independent Methods

**DOI:** 10.1155/2013/420287

**Published:** 2013-08-27

**Authors:** Aurora Rizzi, Elena Crotti, Luigimaria Borruso, Costanza Jucker, Daniela Lupi, Mario Colombo, Daniele Daffonchio

**Affiliations:** ^1^Department of Food, Environmental and Nutritional Sciences (DEFENS), University of Milan, Via Celoria 2, 20133 Milan, Italy; ^2^Faculty of Science and Technology, Free University of Bozen-Bolzano, Piazza Università 5, 39100 Bolzano, Italy

## Abstract

The wood-boring beetle *Anoplophora chinensis* Forster, native to China, has recently spread to North America and Europe causing serious damage to ornamental and forest trees. The gut microbial community associated with these xylophagous beetles is of interest for potential biotechnological applications in lignocellulose degradation and development of pest-control measures. In this study the gut bacterial community of larvae and adults of *A. chinensis*, collected from different host trees in North Italy, was investigated by both culture and culture-independent methods. Larvae and adults harboured a moderately diverse bacterial community, dominated by Proteobacteria, Actinobacteria, and Firmicutes. The gammaproteobacterial family Enterobacteriaceae (genera *Gibbsiella, Enterobacter, Raoultella*, and *Klebsiella*) was the best represented. The abundance of such bacteria in the insect gut is likely due to the various metabolic abilities of *Enterobacteriaceae*, including fermentation of carbohydrates derived from lignocellulose degradation and contribution to nitrogen intake by nitrogen-fixing activity. In addition, bacteria previously shown to have some lignocellulose-degrading activity were detected at a relatively low level in the gut. These bacteria possibly act synergistically with endogenous and fungal enzymes in lignocellulose breakdown. The detection of actinobacterial symbionts could be explained by a possible role in the detoxification of secondary plant metabolites and/or protection against pathogens.

## 1. Introduction

Insects have complex associations with a wide variety of microorganisms. Many bacteria contribute to various physiological functions, including nutrition, development, reproduction, resistance to pathogens, production of pheromones, and immunity [1]. Some symbionts can play essential roles in the insect gut, compensating for diets deficient in certain nutrients or containing recalcitrant organic compounds. For instance, in xylophagous termites the gut microflora enables the host to digest cellulose and fix atmospheric nitrogen [2, 3], and in phytophagous aphids the endocellular symbiont *Buchnera aphidicola* synthesizes essential amino acids that are absent in phloem sap [4, 5]. These gut-microbe interactions are diverse and include antagonism, commensalism, and mutualism and range from obligate to facultative [6]. Obligate symbiotic microorganisms are typically vertically transmitted during early stages of oogenesis or embryogenesis, whereas facultative symbionts can colonize native hosts through horizontal transmission between individuals or acquisition from the diet or the environment [7–11]. All these properties and the important roles that symbionts have in host biology have been proposed for exploitation in novel control strategies of insect pests or for the management of insect-related problems [12–14].

The longhorned beetles (Coleoptera: Cerambycidae) are xylophagous insects which feed on healthy or dead woody plants causing damage of forest and ornamental trees. Many beetles establish a strict association with fungi that naturally colonize their galleries and provide nutrients by lignocellulose degradation and synthesis of other essentials compounds. The genus *Anoplophora* includes xylophagous longhorned beetles, native to eastern Asia, that live on numerous woody plant species. Since its accidental introduction through wood-packing materials and live plants from Asia, it has become an important invasive pest both in Europe and North America. In the United States, the species *A. glabripennis* is spread, whereas in Europe the species *A. chinensis*, form *malasiaca,* is mostly present [15, 16]. The lifecycle of *A. chinensis* lasts 12–24 months, and larvae develop by feeding on cambium, phloem, and subsequently xylem, forming tunnels into the inner bark of the tree and causing death of the host. Oviposition occurs in the bark of the host tree, and eggs, larvae, and pupae can overwinter. In late spring adults emerge and feed on the bark of tender twigs.

Due to their specific diet, comprising highly lignified low-nitrogen wood tissues, gut symbionts may play important roles in the digestive tract of these xylophagous insects, contributing to lignocellulose degradation and synthesis of essential amino acids or vitamins [17]. Studies conducted on larvae of *A. glabripennis* collected in USA and China documented the wide diversity of bacterial taxa harboured in larval guts [18, 19]. The bacterial communities of animals reared on different host trees were extremely variable, with a significant impact on cellulase activity [18]. Larval guts of *A. glabripennis* were also found to be associated with the soft-rot fungus *Fusarium solani*, capable of degrading proteins, cellulose, hemicelluloses, and other woody carbohydrate polymers [20]. However, the recent discovery of an endogenous exocellulase from *A. malasiaca* [21] raises the question of the contribution of gut microorganisms to lignocellulose degradation and, more extensively, their contribution to the beetle's physiology and biochemistry. Further research to characterize the microbial communities of related species, investigating the variation in communities in relation to geography and/or different life stages, could contribute to a better understanding of the complex symbiotic relationships of beetles with microorganisms and the impact of microorganisms on the host lifecycle.

The aim of this study was to investigate the bacterial community associated with both larvae and adults of *A. chinensis*, collected in Italy, using both culture-dependent and independent methods, namely, PCR-DGGE (denaturant gradient gel electrophoresis) and clone library analysis.

## 2. Materials and Methods

### 2.1. Insect Collection and Dissection

Larvae and adults of *A. chinensis* were collected from April to November 2008 at different sites within the infested area in Lombardy, Italy (Table 1). After collection, larvae and adults were maintained separately in sterile containers at 10°C and processed the following day. The insects were surface disinfected with 60% ethanol and rinsed twice in sterile water. Each larva was dissected near a Bunsen burner using sterilized dissection scissors, and the entire gut was extracted from the insect body. The same procedure was followed for adult individuals; in addition, male gonads, and eggs inside the female abdomen were also extracted. The individual guts, gonads and eggs were washed in 4 mL of sterile water, transferred to 1.5 mL tubes with 500 *μ*L of saline, and homogenized using a sterile plastic pestle. Homogenates were used for culture-independent methods and stored at −20°C until use.

### 2.2. Bacteria Isolation

The gut homogenates were 10-fold diluted and directly plated on tryptic soy agar (TSA) and 1/10 strength TSA (Difco, Milan, Italy). Fifty *μ*L of gut homogenates were also used for the enrichment of nitrogen-fixing bacteria in LGI liquid medium (5% sucrose, 0.06% KH_2_PO_4_, 0.02% K_2_HPO_4_, 0.02% MgSO_4_, 0.002% CaCl_2_, 0.001% FeCl_3_, and 0.0002% NaMoO_4_, pH 6 [22]). After growth, the enriched cultures were plated on LGI agar plates containing 20 g/L noble agar (Difco). All media were supplemented with 100 *μ*g/mL cycloheximide. Plates were incubated for 3–5 days at 30°C. The colonies obtained by plating were differentiated based on morphological features including shape, colour, margins, elevation, and texture. Two or more isolates representative of each colony morphology were transferred to fresh agar plates, and pure colonies were stored at −80°C in 15% glycerol.

### 2.3. DNA Isolation, PCR, and Cloning

Total DNA from dissected organs was isolated as previously reported [23]. DNA was extracted by enzymatic and chemical treatment and purified using the Wizard DNA purification resin (Promega, Milan, Italy). PCR amplification of the 16S rRNA gene from bacterial isolates was performed using the universal primers 27F and 1492R [24]. The reaction mixture (50 *μ*L) contained 1× PCR buffer, 1.5 mM MgCl_2_, 0.5 *μ*M of each primer, 0.2 mM of dNTPs, and 1.5 U of Taq DNA polymerase. The DNA template was obtained by transferring a small portion of a pure colony into a PCR tube. The thermal cycling program consisted of 5 min at 95°C, followed by 30 cycles of 45 s at 95°C, 1 min at 55°C, and 1 min at 72°C, with a final extension of 10 min at 72°C.

Two 16 s rRNA gene libraries, one from larvae and one from adults, were constructed using two pooled guts per each library (Table 2). The DNA isolated from the pooled guts was amplified using the primer pair 27F and 1492R, as previously described. The resulting 1.5 kb fragments were cloned into pCRII-TOPO vector (Invitrogen Life Technologies, Milan, Italy) following the manufacturer's protocol. Individual colonies were picked up using sterile pipette tips and used directly for PCR amplification. Insert DNA from 16S rRNA clones was amplified by standard PCR amplification using the primers M13F/M13R [25] and sequenced.

### 2.4. PCR-DGGE Analysis

Bacterial 16S rRNA gene fragments were amplified by PCR using the primer pair GC-357-F/907-R [26–28]. PCR reactions were performed as previously described [29]. Briefly, PCR products (approx. 300 ng) were loaded onto 7% (w/v) polyacrylamide gels (0.75 mm) with a denaturant gradient of 40–60% (100% denaturant contained 7 M urea and 40% formamide). Electrophoresis was run in 1× TAE buffer using a D-Code electrophoresis system (BioRad, Milan, Italy) at 90 V and 60°C for 17 h. Gels were stained with SYBR Green I nucleic Acid Gel Stain (Invitrogen Life Technologies) and documented with GelDoc 2000 apparatus (BioRad) using the Diversity Database software (BioRad). Relevant DNA bands were excised from the gels and eluted in 50 *μ*L of Tris-HCl 10 mM. Five microlitres of DNA was used for 16S DNA fragment reamplification using nonclamped primers and the obtained amplicons sequenced.

### 2.5. Sequencing and Data Analysis

Sequencing of the 16S rRNA gene fragments was performed using the primer 27F at Primm (Milan, Italy). Partial sequences from clones and bacterial isolates were compared against the National Center for Biotechnology Information (NCBI) genomic database with the BLAST (http://blast.ncbi.nlm.nih.gov/Blast.cgi) search alignment tool. A collection of phylogenetically related sequences was obtained from the NCBI database. Sequences from clones were taxonomically classified by the RDP-II Naive Bayesian Classifier (http://rdp.cme.msu.edu/classifier/classifier.jsp) using an 80% confidence threshold. Sequence alignment was carried out and phylogenetic trees constructed using MEGA software, version 5.1 [30]. The trees were constructed using the maximum likelihood algorithm and Tamura Nei parameter correction and were bootstrapped 1000 times.

## 3. Results

### 3.1. Bacterial Community in Larval and Adult Guts

**Figure 1:**
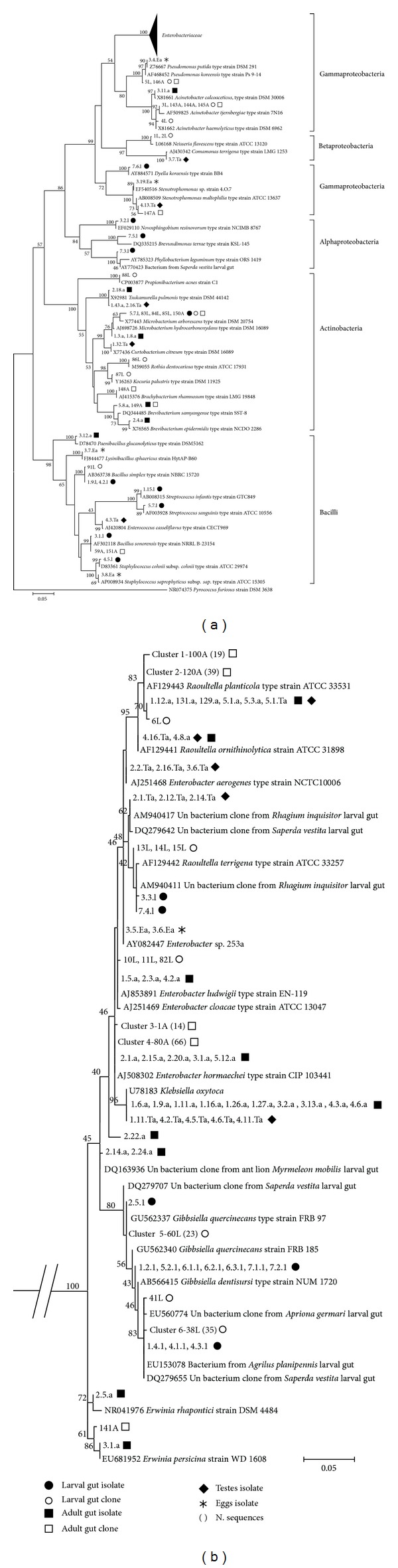
Phylogenetic tree of partial bacterial 16S rRNA sequences retrieved from culturing and clone library. Bacterial sequences fell mainly into five classes (a) and most belonged to the *Enterobacteriaceae* family (b). The category of origin in which each species was identified is indicated by symbols. Groups of sequences are compressed into clusters, and the number of sequences is provided in brackets. “Un” indicates an uncultured bacterium. Numbers at nodes represent bootstrap values and are indicated when values were >40%. The scale bar represents 0.05 substitutions per nucleotide position.

Guts of larvae fed on *Alnus* were investigated by both culturing (seven individual guts) and library clones (pool of two guts). The sequences of bacterial 16S rRNA genes from 23 isolates and 91 clones were obtained. Most isolates were strictly affiliated to the *Gibbsiella* genus (Figure 1 and Table 2). Similarly, using library cloning, the majority of gut-derived clones were represented by the *Gibbsiella* genus (*n* = 58, 63%) and bacteria strictly affiliated to *Gibbsiella* and uncultured clones previously identified in larval guts from other wood-boring beetles (*Agrilus planipennis*, *Saperda vestita,* and *Apriona germari*). The genera *Enterobacter* and *Raoultella* were represented in low proportions (approximately 5 and 1%, resp.).

**Figure 2:**
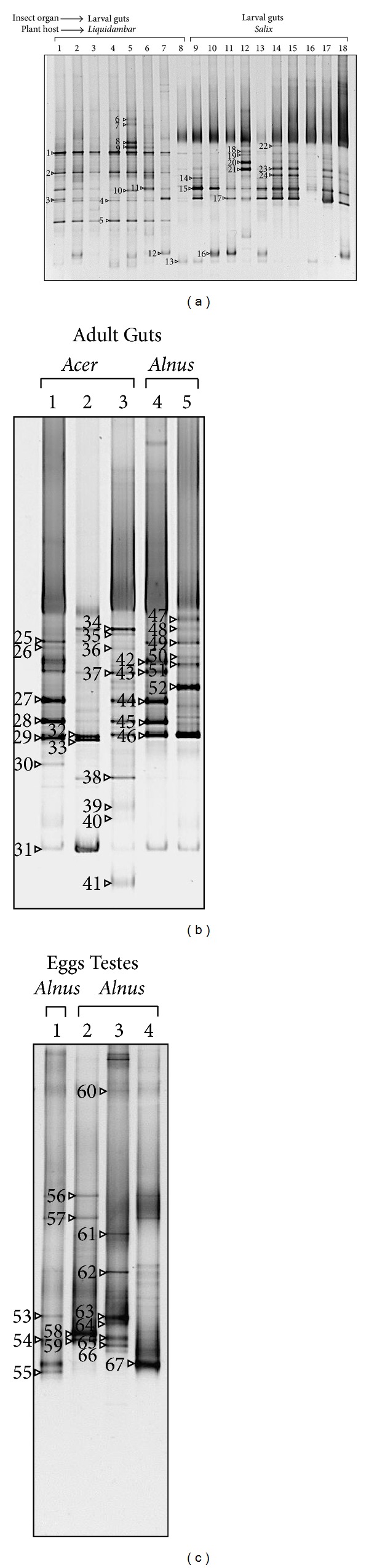
Bacterial DGGE profiles of the 16S rRNA gene PCR products amplified from DNA extracted from guts (a, b) and eggs and testes (b, c) of larvae (a) and adults (b, c) of *A. chinensis* collected from different host trees. Triangles and numbers indicate the bands sequenced (Table 3).

In addition, PCR-DGGE analysis was used to further investigate the dominant microbial species of multiple individuals (Figure 2). The gut bacterial profiles obtained from eighteen larvae grown in *Liquidambar* and *Salix* trees differed markedly but were highly similar to larvae collected from the same site/tree (Figure 2). Sequences of dominant intense bands showing tight affiliationto *Raoultella* (bands 14, 15, 18–21, 23, 24) and unclassified *Enterobacteriaceae *(band 17) were detected in larvae from *Salix*, whereas sequences of faint bands, also related to unclassified *Enterobacteriaceae *(band 4), were found in larvae from *Liquidambar*. *Gibbsiella* (bands 12 and 16) and *Rahnella* (band 11) were occasionally detected, independently of the host tree.

The microbial communities of guts of five adults (four males and one female) fed on *Alnus* or *Acer* were analyzed by both culturing and culture-independent methods. The sequences of 16S rRNA genes from 38 isolates, 151 clones, and 28 DGGE bands were obtained (Tables 2 and 3). Overall, the results of the different analyses indicated that the *Enterobacteriaceae* were the dominant bacteria also in the adult gut. In particular, DGGE analysis, in accordance with culturing, indicated that* Enterobacter* (bands 29, 32, 33, 46) was detected in all five individuals tested, whereas *Klebsiella* (25–28, 42–45) and *Raoultella* (47–51) were found only in some individuals (3 and 1 out of 5 individuals, resp.). One adult individual presented *Enterobacter* (bands 32 and 33) (Figure 2) and microorganisms strictly affiliated to the genus *Erwinia* (Figure 1). Microorganisms affiliated to *Enterobacter* were also identified when performing enrichment of nitrogen-fixing bacteria. Data from library cloning performed on beetles fed on *Alnus* (pool of two guts) indicated an abundance of *Enterobacter* and *Raoultella* in the gut microbial community, with percentages over the total sequenced clones of 52% (*n* = 80) and 38% (*n* = 58), respectively. This result is consistent with the high intensities of the bands relative to these bacteria in DGGE gels. However, no library clones related to the genus *Klebsiella* were detected, maybe due to differences in PCR amplificability of DNA extracted from the two guts and/or to the different amount of template DNA in PCR reactions due to the different sizes of adult guts.

All the analytical methods used revealed a rather diverse community generally characterized by the dominance of *Enterobacteriaceae* in both larvae and adult stages and the occurrence of several species encompassing different taxa (*Proteobacteria*, *Actinobacteria*, *Firmicutes,* and *Bacteroidetes*). In particular, some other Gammaproteobacteria, such as *Acinetobacter* and *Pseudomonas, *were detected in larval and adult guts by culture-independent analyses. Members of Alpha- and Betaproteobacteria groups were found by DGGE and isolation methods in both larvae and adults. Interestingly, *Ralstonia*, *Massilia,* and *Methylobacterium* were found in all larval individuals that fed on *Liquidambar*. It can be speculated that the aromatic resin produced from this host tree species had an impact on the microbial composition of the larval gut communities. The gut bacterial microbiome of *Anoplophora* comprised additional representatives of Actinobacteria and Firmicutes. In particular, the genera *Microbacterium* and *Bacillus* were detected in both larvae and adults using the majority of methods. Some species were detected occasionally in larvae or adults by culture or culture-independent analysis. *Rothia*, *Kocuria*, *Propionibacterium*, *Enterococcus*, *Streptococcus*, *Staphylococcus,* and *Chryseobacterium* were found in larvae, while *Brevibacterium*, *Tsukamurella*, and *Paenibacillus* were found in adults.

### 3.2. Bacterial Community in Adult Testicles and Immature Eggs

Data from isolation and DGGE detection methods revealed an abundance of *Enterobacteriaceae* also associated with testicles and eggs (Table 2 and Figure 2). Similar to microbial gut investigation, the DGGE patterns indicated that the occurrence of diverse microbial genera (*Enterobacter*, *Raoultella*, *Klebsiella,* and *Buttiauxella*) and unclassified bacteria varied among the samples. *Lysinibacillus sphaericus* and *Stenotrophomonas maltophilia* isolates were detected in both testicles and eggs. *Staphylococcus* was also found in eggs, whereas other microorganisms, generally of environmental origin, were identified in testicles.

## 4. Discussion

Overall, the results indicated that the gut microbiota of larvae and adults of *Anoplophora chinensis* was relatively complex being constituted by bacteria placed in six different bacterial classes. A total of 23 and 32 bacterial genera were found in larvae and adults (19 in the gut), respectively, by both culture-dependent and independent methods. This moderately high diversity is in accordance with previous data reported for larval forms of the species *Anoplophora glabripennis* [18, 19]. Twenty-three bacterial taxa were harboured in the larval gut of *A. glabripennis* from China, and a range of 5–31 genera, depending on the host tree, were found in the larval gut of field-collected *A. glabripennis *from USA. The bacterial communities, especially in the case of larvae, showed significant differences as a function of host tree, site of sampling, and, to a lesser extent, specific individuals. The influence of host tree was particularly evident in the case of larvae. Consistent with previous results, the complexity of the bacterial community was higher in larvae fed on the host trees preferred by the insects, which in this study were *Acer* and *Salix.* In addition, we observed that larvae from these trees contained a higher proportion of *Enterobacteriaceae*. According to Geib et al. [18], the plasticity that characterizes the *Anoplophora* bacterial community is probably the reason for the broad host range of this beetle. However, regardless of differences in the insect species analyzed and geographic location of sampling (USA, China, Italy), the bacterial communities found in studies of the larvae of *Anoplophora* spp. are quite similar. Interestingly, the studies investigating the taxonomy and diet of bark-beetles related to *Anoplophora* spp. found the majority of these xylophagous insects to have a lower bacterial diversity than *Anoplophora* spp., ranging from four taxa identified in *Tetropium castaneum* [31] to an average of about ten taxa in *Dendroctonus *species [32–35]. The toxic activity of certain tree chemicals, such as terpenes in pine resin, may be one of the factors determining the relatively scarce species diversity in the gut of these beetles with respect to *Anoplophora*.

In this study, for the first time we showed that the bacterial community was rather conserved also in adults regardless of the shift in diet occurring after the metamorphosis, with the larvae fed on cambium, phloem, and xylem while the adults on foliage and tender bark. An analogous finding was observed in the case of another wood-boring beetle *Agrilus planipennis* [36], which was similar to *Anoplophora* in the complexity of the larval gut community [37].

The observed stability in the composition of the bacterial community at a high taxonomic level may indicate that the overall function of the community is achieved despite variations in its bacterial members. This may indicate that though most symbionts are environmentally-derived transient bacteria, at least some may play a key role in the physiology of this beetle. In particular, the dominance of *Enterobacteriaceae* and Gammaproteobacteria in both larval and adult forms suggests that they are a constant fraction of the gut bacterial community and may be beneficial to host fitness because of their various abilities to hydrolyze and ferment carbohydrates, catalyze nitrogen fixation, and produce vitamins and pheromones. It should be noted that this phylogenetic group of microorganisms has been commonly detected in the gut of diverse insect orders and host diet, with the exception of detritivorous, pollenivorous, and dead wood xylophagous insects [36]. In *Anoplophora*, such microorganisms might act as facultative mutualistic bacteria recurrently acquired during feeding by ingestion and possibly horizontally transmitted between individuals. In particular, the recurrent detection of the diazotrophs *Enterobacter* sp., *Klebsiella* sp., *Raoultella* sp., *Rahnella* sp., and *S. maltophilia* suggests that their contribution to beetle nitrogen requirements may be noteworthy [38], as observed in other insect orders [39]. In addition, considering that no obligate anaerobic bacteria were identified, facultative anaerobic bacteria may work as oxygen scavengers and could have a significant role in creating the microsite anaerobic conditions necessary to allow nitrogen fixation [34]. Members of the *Enterobacteriaceae* are also known to be involved in pheromone production; for example, common gut isolates in locusts, *E. cloacae*, *K. pneumonia,* and *P. agglomerans*, are responsible for the production of components of a locust cohesion pheromone [40].

Interestingly, the presence of Gammaproteobacteria and more generally the composition of bacterial communities, are rather similar in different xylophagous beetles and significantly distinguished from those of insects feeding on dead lignocellulose tissues, such as termites. The diet and, consequently, mechanisms of digestion evolved in the host, including those related to the host gut anatomy, are thought to play an important role in structuring the bacterial community [36]. In view of recent reports identifying a novel endogenous exo/endocellulase from *A. malasiaca*, together with the characteristic anatomy of this beetle which harbours a relatively small hindgut, it is likely that the bacterial community associated with *Anoplophora* spp. is more closely related to host fitness rather than being primarily involved in wood degradation, though several lignocellulose-degrading microbes can be harboured [41, 42]. Considering the bacteria identified in this study, *Pseudomonas putida*, *Kocuria,* and *Acinetobacter* were previously shown to have lignin degradation activity [43, 44]; *Bacillus*, *Paenibacillus*, *Staphylococcus* (Firmicutes), *Sphingomonas* (Alfaproteobacteria), *Ralstonia*, *Comamonas* (Betaproteobacteria), *Dyella ginsengisoli*, *Stenotrophomonas* (Gammaproteobacteria), *Kocuria*, *Brevibacterium* (Actinobacteria), and *Chryseobacterium* (Bacteroidetes) were shown to have cellulose and/or aromatics degradation capabilities [45, 46]. In addition to the bacterial lignocellulose-degrading activities, the nutrient-extracting capacities exerted by fungi strictly associated with the host are thought to contribute to host nutrition, as recently indicated by enzymatic proprieties of the *A. glabripennis* isolate *F. solani* [47]. Moreover, some of these bacteria with specific enzymatic degrading activities are thought to play important roles in the detoxification of plant compounds, production of metabolites against pathogens, and plant-insect interactions [46, 48]. For example, several bacterial genera affiliated to Actinobacteria, Gammaproteobacteria, Betaproteobacteria, and Firmicutes that are contained in the oral secretions of the bark beetle *Dendroctonus rufipennis* were demonstrated to significantly inhibit the growth of antagonistic fungi [49]. In particular, recent findings suggest that symbiotic associations between insects and Actinobacteria could play a crucial role in the protection of the insect host, or its nutritional resources, against parasitoids or predators [50]. In this study, various actinobacterial genera were detected, though they represented a small fraction of the microbiome associated with *Anoplophora* spp.; further research is necessary to elucidate their potential functions.

A preliminary characterization of the bacterial communities associated with testicles and eggs of *Anoplophora chinensis*, despite being limited by the low number of individuals analyzed, allowed us to obtain initial information regarding the microorganisms potentially associated with these organs. Taken together, the results showed that the same microbial species identified in the insect gut were present in these tissues. In accordance with a previous study, it is noteworthy to mention the occurrence of a *Xanthomonadaceae* family member associated with immature eggs that may be vertically transmitted from the mother to the offspring [51].

## 5. Conclusions

The bacterial gut community of *A. chinensis* is relatively diverse and this diversity is maintained throughout different life stages and geographic locations. The community does not appear to be primarily involved in lignocellulose degradation, but conservation of its members at high ranks suggests that these bacteria are beneficial to the host fitness and may contribute to insect nutrition, presumably by providing a fixed nitrogen source. Further studies are needed to elucidate the specific functions of gut-associated bacteria. Similarly, further investigation is necessary to clarify the role and mode of transmission of bacteria associated with the reproductive systems of *Anoplophora* spp.

## Figures and Tables

**Table 1:** *Anoplophora chinensis* collection and detection strategies used in this study.

Host tree	Insects (no.)	Detection strategies
*Alnus *	Larvae (9)	Isolation and library clones (pool of 2 guts)
*Liquidambar *	Larvae (8)	DGGE
*Salix caprea *	Larvae (10)	DGGE
*Acer saccharinum *	Adults (3)	Isolation and DGGE
*Alnus *	Adults (2)	Isolation, library clones (pool of 2 guts), and DGGE

**Table 2:** Bacterial taxa identified in larvae and adults of *Anoplophora chinensis* by culture and culture-independent methods.

Phylum	Class	Family	Genus^a^	Larval gut			Adult gut			Testicles and eggs^d^	
				Detection strategy			Detection strategy			Detection strategy	
				Isolation (al)^c^	Library (al)^c^	DGGE^b^ (li, sa)^c^	Isolation (ac, al)^c^	Library (al)^c^	DGGE^b^ (ac, al)^c^	Isolation (ac, al)^c^	DGGE^b^ (ac, al)^c^
Proteobacteria	Alfaproteobacteria	Caulobacteraceae	*Brevundimonas *	1			1 ac		ac		
		Sphingomonadaceae	*Sphingomonas *						ac		
			*Novosphingobium *	1							
		Phyllobacteriaceae	*Unclassified *	1							
		Methylobacteriaceae	*Methylobacterium *			li			ac		
	Betaproteobacteria	Comamonadaceae	*Comamonas *							1T ac	
		Neisseriaceae	*Neisseriae *		2						
		Oxalobacteraceae	*Massilia *			li			ac		
			*Ralstonia *			li			ac		
	Gammaproteobacteria	Enterobacteriaceae	*Enterobacter *		4		3 ac, 6 al*	80	ac, al	3T ac, 3E ac	E ac, T ac, al
			*Klebsiella *				8 ac, 2 al		ac, al	1T ac, 4T al	E ac, T ac, al
			*Raoultella *		1	sa	5 ac, 1 al	58	ac, al	3T al	E ac, T ac, al
			*Gibbsiella *	7	58	li, sa					
			*Rahnella *			li					
			*Erwinia *				2 ac	1			
			*Buttiauxella *								T ac
			*Unclassified *	3	16	li, sa	2 ac	2	ac	3T ac	T ac
		Xanthomonadaceae	*Stenotrophomonas *					1		1T al, 1E ac	
			*Dyella *	1							
		Sinobacteraceae	*Nevskia *						ac		
		Pseudomonadaceae	*Pseudomonas *		1	sa		1		1E ac	
		Moraxellaceae	*Acinetobacter *		2		1 ac	3			

Actinobacteria	Actinobacteria	Microbacteriaceae	*Microbacterium *	1	3	li, sa	2 ac	1	ac		
			*Curtobacterium *							1T ac	
		Dermabacteraceae	*Brachybacterium *					1			
		Brevibacteriaceae	*Brevibacterium *				1 ac, 1 al	1			
		Propionibacteriaceae	*Propionibacterium *		1						
		Micrococcaceae	*Rothia *		1						
			*Kocuria *		1						
		Tsukamurellaceae	*Tsukamurella *				2 ac			1T ac	

Firmicutes	Bacilli	Bacillaceae	*Bacillus *	3	1			2			
		Paenibacillaceae	*Paenibacillus *				1 ac				
		Enterococcaceae	*Enterococcus *			li				1T al	
		Streptococcaceae	*Streptococcus *	2							
		Staphylococcaceae	*Staphylococcus *	1						1E ac	
		Planococcaceae	*Lysinibacillus *							1E ac	

Bacteroidetes	Flavobacteriia	Flavobacteriaceae	*Chryseobacterium *	2							
	Sphingobacteriia	Chitinophagaceae	*Chitinophaga *								T ac

^a^Identification based on NCBI (95% limit) and RDP Classifier (80% confidence threshold) results is given, when possible, at genus level.

^b^Individuals positive for the presence of the specific band in the DGGE analysis.

^c^The host tree from which insects were sampled are indicated in brackets: ac: *Acer*; al: *Alnus*; li: *Liquidambar*; sa: *Salix*.

^d^T: testicles; E: immature eggs.

*1 out of 3 ac, and 3 out of 6 al were recovered on LGI medium.

**Table 3:** Closest relatives of bacterial 16S rRNA gene sequences of DGGE bands obtained from larvae and adults of *Anoplophora  chinensis*.

Band	Closest relative (accession no.)	Identity (%)	Bacterial division
1	*Ralstonia solanacearum* (JQ655458)	100	Betaproteobacteria
34	*Ralstonia* sp. (JN714979)	99.1–99.7	Betaproteobacteria
2	Uncultured *Massilia* sp. (EF075289)	99.3	Betaproteobacteria
35	Uncultured *Massilia* sp. (JN648276)	99.3	Betaproteobacteria
37	*Massilia* sp. (AB623119)	98.4	Betaproteobacteria
3, 17	*Raoultella terrigena* (AY292875)	100	Gammaproteobacteria
	*Enterobacter* sp. (AB673457)	99.2–99.6	Gammaproteobacteria
14, 15, 23, 24	Uncultured *Raoultella* sp. (FJ467399)	99.2–99.8	Gammaproteobacteria
	*Raoultella terrigena* (JN815233)	99.0–99.6	Gammaproteobacteria
18, 19, 20, 21	*Raoultella ornithinolytica* (HE578796)	98.4–99.8	Gammaproteobacteria
47, 48, 49, 50, 51	*Raoultella planticola* (JN835545)	99.2–99.6	Gammaproteobacteria
52	*Raoultella ornithinolytica* (HQ242732)	98.4	Gammaproteobacteria
53	*Raoultella planticola* (HE610795)	99.6	Gammaproteobacteria
56	Uncultured* Raoultella* sp. (FJ467399)	99.6	Gammaproteobacteria
58	*Raoultella terrigena* (GQ169108)	100	Gammaproteobacteria
62	*Raoultella ornithinolytica* (HQ242729)	99.8	Gammaproteobacteria
10	Gammaproteobacterium (EF111244)	99.8	Gammaproteobacteria
11	*Rahnella* sp. (JQ864392)	99.7	Gammaproteobacteria
12, 16	*Gibbsiella dentisursi* (AB566415)	99.7	Gammaproteobacteria
22	*Pseudomonas* sp. (JQ522968)	99.0	Gammaproteobacteria
25, 42, 45, 28	*Klebsiella* sp. (GU301269)	99.6–99.8	Gammaproteobacteria
27, 44, 43, 26	*Klebsiella oxytoca* (JF772070)	99.4–99.8	Gammaproteobacteria
54, 59	*Klebsiella *sp. CPK (GU301269)	99.6	Gammaproteobacteria
57	*Klebsiella oxytoca* (JX196648)	99.2	Gammaproteobacteria
29, 46	*Enterobacter* sp. JJDP1 (JQ726698)	100	Gammaproteobacteria
32	*Enterobacter* sp. ZYXCA1 (JN107752)	100	Gammaproteobacteria
33	*Enterobacter* sp. IICDBZ6 (JN836923)	99.8	Gammaproteobacteria
55	*Enterobacter ludwigii* (KC139450)	98.7	Gammaproteobacteria
67	*Enterobacter *sp. (JN129489)	98.9	Gammaproteobacteria
36	*Nevskia* sp. (JQ710439)	100	Gammaproteobacteria
63	*Enterobacteriaceae *bacterium (HM235485)	99.8	Gammaproteobacteria
65	*Buttiauxella *sp. (JF281151)	99.8	Gammaproteobacteria
66	*Buttiauxella *sp. (JX406856)	99.8	Gammaproteobacteria
4	*Methylobacterium* sp. (FJ225120)	100	Alphaproteobacteria
5, 38	*Methylobacterium populi* (JQ660234)	99.7–100	Alphaproteobacteria
39	*Brevundimonas* sp. S2U9 (HE814668)	100	Alphaproteobacteria
40	*Sphingomonas* sp. D40y (HE962513)	100	Alphaproteobacteria
6, 8	*Enterococcus* sp. (JF813181)	99.0–100	Firmicutes
7	*Enterococcus gallinarum* (JQ805717)	100	Firmicutes
9	*Enterococcus casseliflavus* (JX035954)	100	Firmicutes
61	*Lysinibacillus sphaericus* (JN377788)	98.7	Firmicutes
13, 41	*Microbacterium* sp. (EU584504)	100	Actinobacteria
30	Uncultured bacterium (JN394024)	99.8	Unclassified
64	Uncultured bacterium (GQ411142)	99.6	Unclassified
60	Uncultured* Chitinophaga* sp. (KC110981)	100	Bacteroidetes
31	Uncultured plastid (HM270514)	100	Eucariote plastid
